# Metabolic alterations and potential biomarkers in unstable angina investigated by lipidomic analysis

**DOI:** 10.3389/fmolb.2026.1833340

**Published:** 2026-05-22

**Authors:** Nan Feng, Linhe Wang, Guoan Zhao, Yanlin Zhou

**Affiliations:** 1 The First Affiliated Hospital of Xinxiang Medical University, Xinxiang, China; 2 North Henan Medical University, Xinxiang, China

**Keywords:** biomarkers, lipidomic analysis, machine learning, unstable angina, UPLC-MS/MS

## Abstract

**Introduction:**

Unstable angina (UA) represents a critical condition within the broader context of acute coronary syndromes, characterized by episodes of sudden chest pain resulting from insufficient blood flow to the myocardium. The pathophysiological mechanisms underlying UA remain complex and poorly understood, necessitating further investigation into the metabolic alterations associated with this condition. Identifying specific biomarkers for UA is crucial for improving diagnostic accuracy and facilitating timely therapeutic interventions.

**Methods:**

The present study was designed to elucidate the metabolic profile of UA by enrolling 60 patients diagnosed with UA, alongside 60 healthy controls. Participants were stratified into discovery and validation cohorts, with each group comprising 30 UA patients and 30 controls. The diagnosis of UA was performed in accordance with the 2020 European Society of Cardiology guidelines and the 2019 Chinese clinical pathway for UA management. Blood samples were meticulously collected following an overnight fast, processed, and subsequently stored at −80 °C. A comprehensive lipidomic analysis was executed utilizing ultra-high-performance liquid chromatography-tandem mass spectrometry (UPLC-MS/MS), with compound identification referenced from the Metware Database. Data analysis involved advanced multivariate statistical techniques, including principal component analysis and orthogonal partial least squares-discriminant analysis.

**Results:**

The analysis revealed significant metabolic discrepancies between the UA and control groups, identifying a total of 193 differential metabolites, of which 67 were notably upregulated in the UA cohort. Pathway enrichment analysis highlighted critical alterations in glycerophospholipid metabolism and necroptosis pathways. Furthermore, the application of machine learning algorithms, specifically neural networks and random forests, enabled the identification of key metabolic biomarkers, including TxB3, LPC(16:0/0:0), and DL-Carnitine, which exhibited robust diagnostic potential.

**Conclusion:**

In summary, our findings indicate that these metabolites may serve as promising candidate diagnostic biomarkers for UA, providing valuable insights into its pathophysiology.

## Introduction

Unstable angina (UA) is a critical clinical subtype within acute coronary syndrome (ACS), distinguished by its acute onset, rapid progression, and elevated mortality risk (Byrne et al., 2023). As a significant component of ACS, UA, along with other manifestations such as acute myocardial infarction, constitutes sudden cardiovascular events. Failure to promptly recognize and intervene in these conditions can result in severe clinical consequences. Therefore, early and accurate diagnosis is essential for reducing mortality and improving patient outcomes. Currently, serum high-sensitivity troponin (hs-cTn) is the primary diagnostic marker for non-ST-segment elevation ACS (NSTE-ACS). Nevertheless, this biomarker exhibits limitations in sensitivity during the initial symptomatic phase, resulting in a diagnostic ambiguity for certain patients and posing challenges for the early confirmation of UA (Wu et al., 2025; Bhatt et al., 2022). Additionally, conventional imaging assessments and clinical symptom analysis have certain shortcomings, potentially leading to diagnostic delays or misjudgments that hinder the timely development of treatment strategies (Cholack et al., 2022).

**TABLE 1:** Patient characteristics and baseline clinical details.

Characteristic		Discovery cohort		Validation cohort	
		HC1	UA1	HC2	UA2
Sex	Male	16	17	15	16
	Female	14	13	15	14
Age	≤60	17	15	16	14
	>60	13	15	14	16

**FIGURE 1:**
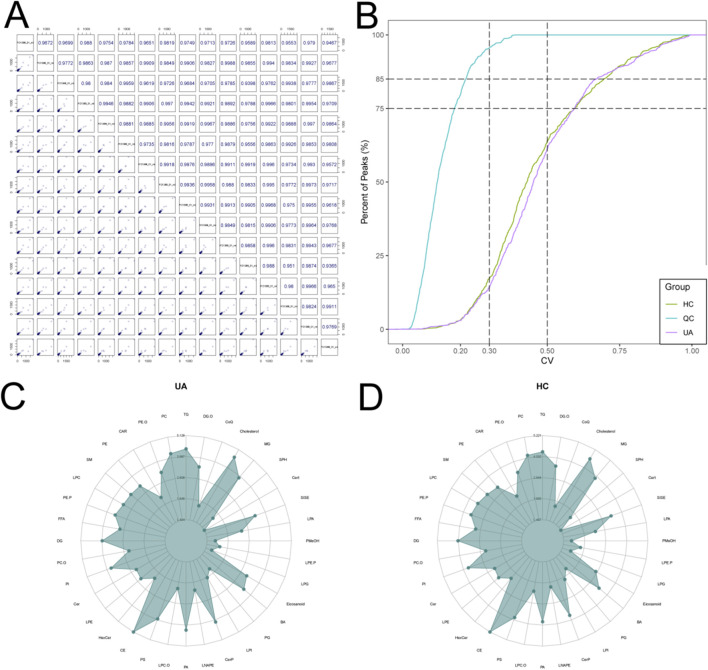
Sample Quality Control and Lipid Composition Analysis. **(A)** Correlation analysis of quality control (QC) samples. Diagonal grids display sample names; scatter plots in the lower left show metabolite abundances between QC samples, with each point representing a metabolite; the upper right grids indicate Pearson correlation coefficients. **(B)** Distribution of coefficients of variation (CV) for QC samples. The x-axis represents CV values, and the y-axis indicates the proportion of metabolites with a CV lower than the corresponding threshold. **(C)** Radar chart illustrating the relative proportions of different lipid classes in the UA group. **(D)** Radar chart showing the relative proportions of different lipid classes in the HC group.

**FIGURE 2:**
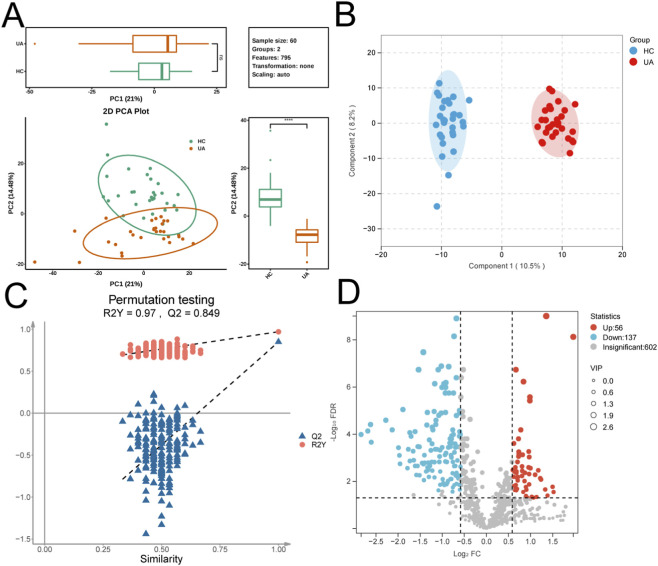
Screening of Differential Metabolites. **(A)** Principal component analysis (PCA) of metabolic profiles between the UA and HC groups. Each point represents a sample, and the distance between points reflects inter-sample similarity. **(B)** Orthogonal projections to latent structures-discriminant analysis (OPLS-DA) score plot for the UA and HC groups. Points represent samples colored by group, and ellipses indicate 95% confidence intervals. **(C)** Permutation test plot of the OPLS-DA model. The x-axis represents the correlation between permuted and original groups, and the y-axis shows the values of R^2^Y and Q^2^. **(D)** Volcano plot of differential metabolites.

The pathological mechanisms underlying coronary atherosclerosis and plaque formation are intricately linked to disruptions in lipid metabolic homeostasis. Dyslipidemia facilitates the deposition and oxidative modification of low-density lipoprotein (LDL) within the vascular wall, marking the initial stage of atherosclerotic plaque development. Simultaneously, it induces endothelial dysfunction and triggers sustained inflammatory responses, collectively driving plaque progression towards instability and increasing the risk of acute coronary events (Kraler et al., 2025). Mechanistically, dyslipidemia exacerbates vascular damage through multiple pathways, including direct endothelial cell dysfunction due to lipid overload, stimulation of inflammatory mediator release, and promotion of immune cell activation and infiltration (Islam et al., 2024; Trakaki and Marsche, 2021). Importantly, elevated plasma levels of proprotein convertase subtilisin/kexin type 9 (PCSK9) significantly advance atherosclerosis progression by modulating lipid metabolism and amplifying inflammatory responses (Sotler and Sebestjen, 2023). Furthermore, dyslipidemia adversely affects both vascular and lymphatic endothelial function, thereby intensifying local inflammation and altering the metabolic microenvironment (Cifarelli et al., 2022; Gonzalez-Nieves et al., 2025). These complex pathological changes collectively promote the formation of coronary atherosclerotic plaques and increase their susceptibility to rupture, forming a critical pathological foundation for acute coronary syndromes, such as unstable angina.

Metabolomics, an integral aspect of systems biology, facilitates the systematic, high-throughput analysis of metabolites within biological systems. This methodology uncovers metabolic signature profiles associated with disease states, identifies potential biomarkers, and provides innovative insights for early disease detection and risk stratification (Lin et al., 2024). Utilizing this technology, research has identified lipids and related metabolites that show differential expression across various subtypes of ACS and have predictive value for adverse clinical outcomes (Yang et al., 2023). Multimodal models that integrate metabolomic biomarkers with clinical indicators have demonstrated significant capabilities in disease discrimination and prediction, thereby establishing a foundation for the advancement of precision diagnostics and personalized interventions (Liu et al., 2021; Cui et al., 2025).

Building on this foundation, the current study proposes the use of ultra-performance liquid chromatography-tandem mass spectrometry (UPLC-MS/MS) technology to perform lipid metabolomic profiling in patients with unstable angina and healthy controls. This approach aims to systematically elucidate the critical role of lipid metabolic dysregulation in the pathogenesis of the disease. Additionally, machine learning algorithms will be utilized to identify and validate metabolic biomarkers for the early detection of unstable angina. This study seeks to provide novel theoretical insights and methodological support for the early diagnosis, risk prediction, and optimization of treatment strategies for unstable angina, thereby advancing its clinical management towards precision medicine.

## Materials and methods

### Study population inclusion

This investigation included a total of 120 participants, comprising 60 patients diagnosed with UA and 60 healthy control (HC) undergoing routine medical examinations. The study population was stratified into two cohorts: a discovery cohort and a validation cohort, each consisting of 30 UA patients and 30 HC (Table 1). The diagnosis of UA was conducted in accordance with the 2020 European Society of Cardiology (ESC) Guidelines for the Management of Non-ST-Elevation Acute Coronary Syndromes, as well as the Chinese Clinical Pathway for Interventional Treatment of Unstable Angina Pectoris (2019). Informed consent was obtained from all participants, and the study protocol was approved by the relevant ethical committee.

### Blood sample collection

All blood samples from patients with UA in this study were obtained immediately upon their presentation to the emergency department or chest pain center with acute chest pain. This was conducted following the clinical diagnosis of UA and prior to any significant invasive procedures, such as coronary angiography or revascularization. Blood samples, approximately 5 mL in volume, were collected from all participants following an 8-h fasting period. The samples were centrifuged within 30 min of collection, and the supernatant was isolated and stored at −80 °C.

### Sample pre-processing

Serum samples were initially thawed in an ice bath following their retrieval from storage at −80 °C. Once thawed, the samples underwent vortexing for 10 s and were subsequently centrifuged at 4 °C and 3,000 rpm for a duration of 5 min. A volume of 50 μL from each sample was then transferred to a labeled centrifuge tube. To this, 1 mL of a lipid extraction solution containing an internal standard mixture was added. The mixture was vortexed for 2 min and subjected to sonication for 5 min. Subsequently, 200 μL of distilled water was added, followed by additional vortexing for 1 min. The samples were then centrifuged at 4 °C and 12,000 rpm for 10 min. Post-centrifugation, 200 μL of the supernatant was transferred to a labeled centrifuge tube for concentration and drying. The dried residue was resuspended in 200 μL of a lipid reconstitution solution. This final solution was prepared for analysis *via* LC-MS/MS. Throughout the process, low temperatures were maintained to ensure the stability of the samples.

### Lipidomics analysis and data processing

This study employed a quantitative lipid metabolomics platform, diverging from the traditional non-targeted metabolomics approach. The primary distinction lies in our use of over 60 isotopic internal standards, enabling the quantification of 795 lipids through standard curves. The lipidomics analysis was conducted using an UPLC-MS/MS system, which included an ExionLC™ AD UPLC and a QTRAP® 6,500+ triple quadrupole mass spectrometer (SCIEX). Compound identification was based on the laboratory’s proprietary Metware Database (MWDB), which facilitated the qualitative analysis of detected lipid molecules by comparing retention times and mass spectrometry fragmentation patterns. Quantitative analysis was performed using the multiple reaction monitoring (MRM) mode. In this mode, the first quadrupole selected precursor ions (parent ions) corresponding to target lipids, thereby eliminating interference from non-target molecular ions. The selected ions then underwent collision-induced dissociation in the collision cell to produce characteristic fragment ions. The third quadrupole further screened specific fragment ions, thereby significantly enhancing detection specificity and sensitivity through dual ion selection. Following data acquisition, chromatographic peaks corresponding to each target lipid are integrated. Matrix effects are addressed using an internal standard method to determine absolute concentrations. This methodology provides high selectivity, excellent reproducibility, and precise quantification, rendering it suitable for high-throughput absolute quantification of lipid molecules in complex biological samples. A standard curve equation is established through a linear regression model correlating standard concentrations with response values. The peak area ratio of experimental samples is then applied to this equation, incorporating correction factors such as dilution factors, to ultimately calculate the absolute content of target metabolites within the sample. The entire analytical process rigorously adheres to quality control standards, including the regular analysis of quality control samples, monitoring of instrument stability, and evaluation of method reproducibility. The resulting data undergo multi-tiered validation and statistical analysis to ensure the accuracy and reliability of the analytical results.

### Multivariate statistical analysis

Principal Component Analysis (PCA) was performed using the stats package, an unsupervised dimensionality reduction technique to identify primary sources of variation and natural clustering tendencies within the data. The results were visualized using the ‘ggplot2′ package, which facilitated the creation of PCA score plots to intuitively depict sample distributions and potential outliers. Additionally, to enhance category discrimination and identify variables with inter-group discriminative power, (Orthogonal) Partial Least Squares Discriminant Analysis (OPLS-DA) was employed using the ‘ropls’, ‘base’, and ‘stats’ packages. This supervised method optimizes inter-group differences by incorporating sample category information, thereby effectively extracting variation pertinent to experimental conditions. Concurrently, univariate statistical analyses were performed, including the calculation of fold changes and the application of t-tests, to assess differences in expression levels and statistical significance across groups for each variable. To assess the reliability of the OPLS-DA model and mitigate the risk of overfitting, permutation tests were employed. The results of these tests were visualized using ggplot2, with the x-axis representing the number of permutations and the y-axis depicting the distribution of corresponding statistics (R^2^Y, Q^2^) derived from the permutation model. This visualization facilitated an intuitive evaluation of model significance by allowing for a comparison between these values and the actual statistics obtained from the original model.

### Differential metabolite identification and enrichment analysis

Differential metabolites were screened using criteria of FC > 1.5 or <0.6, VIP >1, and false discovery rate (FDR) < 0.05. Subsequently, KEGG database mapping was employed for ID assignment, and pathway enrichment analysis was conducted using the clusterProfiler package to systematically elucidate their biological functions.

### Machine learning

Multiple machine learning algorithms were employed to construct diagnostic models, aiming to develop stable predictive tools with robust generalisation capabilities. The algorithms employed included neural networks (NNET), support vector machine (SVM), random forests (RF), least absolute shrinkage and selection operator (LASSO), decision trees (DT), and k-nearest neighbors (KNN). During the model training phase, essential preprocessing and feature engineering were conducted on the dataset, and hyperparameters were optimized through cross-validation to enhance the performance of each algorithm. To systematically evaluate and compare the diagnostic efficacy of the models, two complementary assessment strategies were implemented. First, the reverse cumulative distribution of residuals was employed to visually assess model fitting quality and error distribution characteristics across the entire dataset. Second, receiver operating characteristic (ROC) curves were plotted, and the area under the curve (AUC) was calculated to provide a comprehensive and quantitative evaluation of the models’ classification performance and clinical diagnostic potential. The code for the six machine learning methods is provided in Supplementary 1.

### Statistical analysis

The statistical analyses and data visualization for this study were conducted using the R programming language, version 4.2.1. For continuous variables that satisfied the assumptions of normality and homogeneity of variance, independent or paired t-tests were employed for between-group comparisons. In instances where data did not meet the conditions for parametric testing, non-parametric tests, such as the Wilcoxon signed-rank test, were utilized. The significance threshold was established at α = 0.05, indicating that differences were deemed statistically significant when p-values were less than 0.05. Pearson correlation analysis was applied to evaluate the correlations among quality control samples. Additionally, the Empirical Cumulative Distribution Function (ECDF) was used to analyze the frequency of coefficient of variation (CV) values for substances that fell below the reference value.

## Results

### Sample quality control analysis

To ensure the reliability of the data analysis, this study initially performed a systematic quality control (QC) analysis on QC samples. The correlation analysis of these QC samples demonstrated that the correlation coefficients among all samples exceeded 0.9, indicating excellent repeatability and stability in the instrumental detection process, thereby ensuring high overall data quality (Figure 1A). To further evaluate data dispersion, the CV was calculated for each substance, defined as the ratio of the standard deviation to the mean. The CV value effectively reflects data fluctuation during repeated measurements. Analysis of the CV distribution using the ECDF revealed that over 85% of the compounds exhibited CV values below 0.3 (Figure 1B). This finding indicates that the measured values for the vast majority of metabolites were concentrated and demonstrated good reproducibility, further confirming the stability of the experimental workflow. Furthermore, radar charts were employed to visually compare the variations in lipid metabolite content between the two sample groups (Figures 1C,D). The results demonstrated broadly consistent patterns in metabolite composition across the groups, thereby indicating overall experimental consistency and confirming the data’s suitability for subsequent differential analysis.

### Identification of differential metabolites

To systematically discern metabolic variations between the two sample groups, PCA was initially conducted on the 795 detected metabolite features. The analysis revealed a distinct separation trend along the second principal component (PC2), signifying inherent differences in the metabolic profiles between the groups (Figure 2A). Subsequently, supervised OPLS-DA was utilized to enhance the differentiation between groups (Figure 2B). The model evaluation metrics indicated high explanatory power (R^2^Y = 0.97) and predictive efficacy (Q^2^ = 0.849) (Figure 2C). Permutation tests further verified that the model was free from overfitting and demonstrated robust stability. Based on multiple criteria, including FC, VIP, and t-test p-values, a total of 193 significantly differentially expressed metabolites were identified (Figure 2D). Among these, 56 metabolites exhibited significantly upregulated expression in the UA group, whereas 137 displayed higher abundance in the HC group.

### Enrichment analysis

**FIGURE 3:**
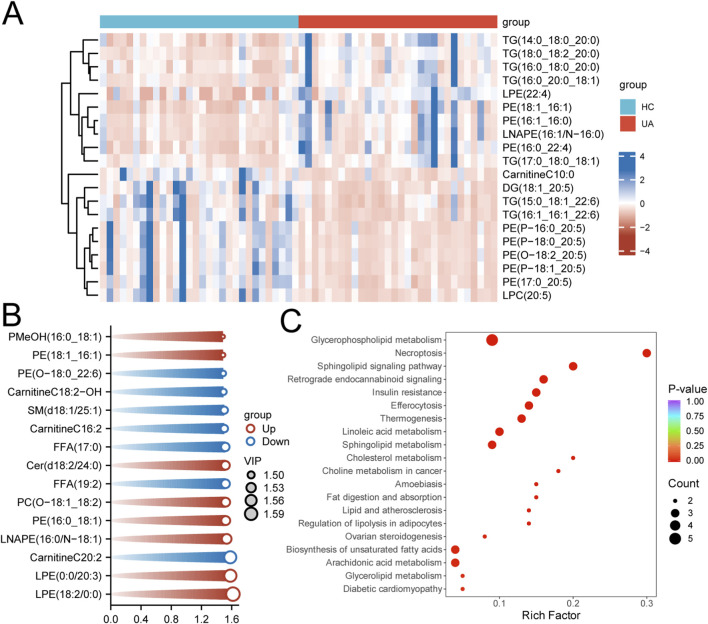
Key Differential Metabolites and Enrichment Analysis. **(A)** Heatmap of the top 20 most significantly altered metabolites ranked by fold change (FC). **(B)** Top 15 most significant metabolites based on variable importance in projection (VIP) scores from the OPLS-DA model. **(C)** KEGG pathway enrichment analysis of all differential metabolites.

A heatmap visually presented the 20 metabolites exhibiting the most pronounced expression differences between groups, with colour gradients clearly illustrating their relative abundance and variation patterns across samples (Figure 3A). Concurrently, to identify metabolites contributing most to inter-group differentiation, the top 15 metabolites were selected based on VIP values and visualised (Figure 3B). These metabolites exhibited high discriminative weights within the (O)PLS-DA model. Subsequently, metabolic pathway enrichment analysis was conducted on all identified differentially expressed metabolites. Results indicated significant enrichment in pathways including glycerophospholipid metabolism, necroptosis, sphingolipid signalling, and retrograde endocannabinoid signalling (Figure 3C).

### Identification of potential metabolic biomarkers

**FIGURE 4:**
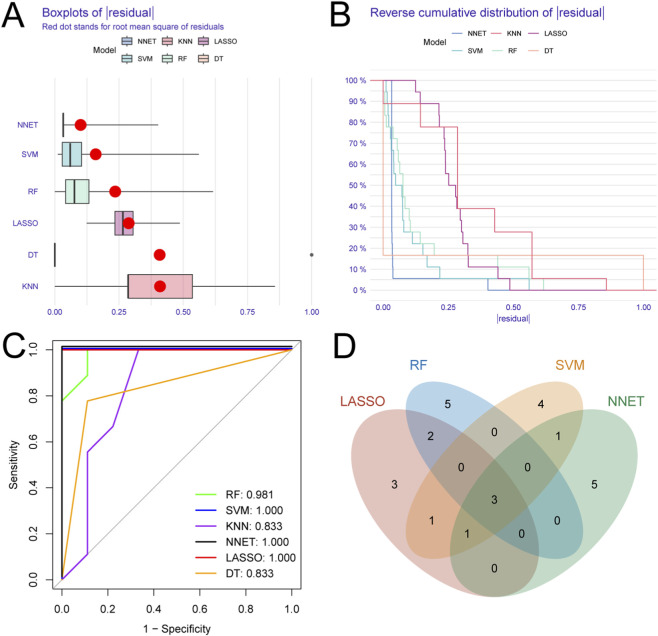
Identification of Potential Metabolic Biomarkers. **(A)** Boxplots displaying the residual distribution of different machine learning models. **(B)** Reverse cumulative distribution of residuals for each machine learning model. **(C)** Receiver operating characteristic (ROC) analysis of different machine learning models. **(D)** Venn diagram showing the overlap of the top 10 candidate biomarkers identified by four machine learning methods: LASSO, random forest (RF), support vector machine (SVM), and neural network (NNET).

In order to investigate potential associations between metabolic dysregulation and the pathogenesis of uric acid, six machine learning algorithms were employed to screen 193 differentially expressed metabolites for the identification of key metabolic biomarkers. The predictive performance of the models was rigorously assessed through analysis of the absolute residual distribution and the AUC, with results illustrated through box plots (Figures 4A,B). The analysis indicated significant prediction errors and relatively low AUC values (with a minimum of 0.833) for the KNN and DT models, leading to their exclusion from further analyses (Figure 4C). Following the removal of these two algorithms, we cross-referenced the features selected by the remaining four machine learning methods. For each algorithm, we identified the top 10 most important metabolites using the algorithm’s intrinsic feature importance metrics. A Venn diagram was utilized to visualize the metabolites identified in common by the different algorithms (Figure 4D). This integrated approach successfully highlighted three consistently pivotal metabolites: TxB3, LPC(16:0/0:0), and DL-Carnitine.

**FIGURE 5:**
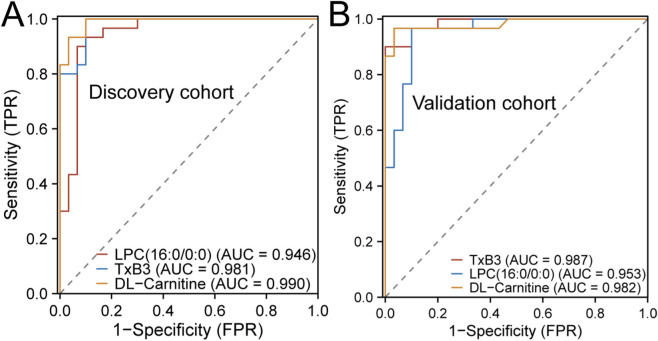
Diagnostic Performance of Potential Biomarkers. **(A)** ROC curve analysis of candidate biomarkers in the discovery cohort. **(B)** ROC analysis of the identified biomarkers in the validation cohort.

### Analysis of the predictive efficacy of potential biomarkers

To systematically assess the diagnostic potential of three key metabolites as biomarkers, ROC curve analysis was performed on both the discovery cohort and an independent validation set. In the discovery cohort, the AUC values for TxB3, LPC(16:0/0:0), and DL-Carnitine were 0.981, 0.946, and 0.990, respectively, demonstrating excellent discriminatory capability for all three metabolites (Figure 5A). To further confirm their robustness and generalizability, the analysis was repeated in the independent validation cohort, resulting in AUC values of 0.987, 0.953, and 0.982, respectively, which were highly consistent with those obtained in the discovery cohort (Figure 5B).

## Discussion

UA constitutes a critical clinical manifestation of acute coronary syndrome, predominantly characterized by transient chest pain resulting from myocardial hypoperfusion (Kovacevic et al., 2025). This condition generally arises due to thrombosis following the rupture of an atherosclerotic plaque, which leads to partial or complete occlusion of the coronary artery. The clinical presentation of UA is notably heterogeneous, ranging from angina occurring at rest to persistent, severe chest pain that may be provoked by minimal exertion (Bhatt et al., 2022; Kite et al., 2022). The pathophysiological mechanisms underlying UA involve intricate interactions among lipid metabolism disorders, inflammatory responses, and endothelial dysfunction, which collectively undermine coronary plaque stability. Given the potential for rapid progression to myocardial infarction, prompt diagnosis and effective intervention are imperative for enhancing patient outcomes.

This study employed a lipidomics approach to systematically compare metabolic differences between UA patients and healthy controls. The cohort comprised 60 subjects: 30 patients meeting clinical diagnostic criteria for UA and 30 healthy controls. All samples underwent standardised collection and processing, with pre-treatment completed under stringent quality control. Lipidomic analysis was performed using UPLC-MS/MS. Ultimately, 795 lipid molecules were identified, with 193 exhibiting significant abundance differences between groups. Differentially expressed metabolites were predominantly enriched in glycerophospholipid metabolism, necrotic apoptosis, and sphingosine signalling pathways. Glycerophospholipid metabolism plays a pivotal role in maintaining cell membrane structural integrity and participating in intracellular signal transduction; abnormalities in this pathway are closely associated with the development of multiple cardiovascular diseases. Recent studies indicate that phospholipid metabolic remodelling holds significant importance in the pathogenesis of UA, suggesting that glycerophospholipid metabolic disorders may be associated with inflammatory activation and endothelial dysfunction in UA patients (Fan et al., 2016; Calderon-Santiago et al., 2014). The findings of this study support the development of intervention strategies targeting this pathway, aiming to restore lipid homeostasis and delay the progression of UA. Necrotic apoptosis, as a form of programmed necrosis, plays a crucial role in cardiovascular diseases. Its process is accompanied by inflammatory responses and tissue damage, constituting one of the key mechanisms in the development of unstable angina. Recent studies indicate that necrotic apoptosis can exacerbate myocardial injury and promote adverse cardiac remodelling following ischaemia (Hu et al., 2019). This study identified pathway enrichment, suggesting necrotic apoptosis may serve as a potential biomarker for UA while providing a basis for exploring its mechanisms and therapeutic value. The sphingolipid signalling pathway exerts crucial regulatory effects on apoptosis, inflammation, and proliferation. Metabolic abnormalities within this pathway are closely associated with multiple cardiovascular diseases, including UA. Bioactive lipids such as sphingosine and 1-phospho-sphingosine have been demonstrated to modulate inflammation and endothelial function (Wang et al., 2023). The significant enrichment of this pathway in our study indicates that sphingolipid metabolites hold promise as novel biomarkers for UA diagnosis and therapeutic targets, with their specific mechanisms warranting further investigation.

Machine learning has demonstrated unique computational advantages in the screening and validation of disease biomarkers, and have been extensively applied in omics research such as metabolomics in recent years (Xu et al., 2024; Guo et al., 2025). In this study, we comprehensively employed six machine learning algorithms to screen three biomarkers with high diagnostic potential from lipid metabolites: TxB3, LPC (16:0/0:0), and DL-carnitine. TxB3, a thromboxane metabolite, plays a well-established role in platelet activation and vasoconstriction regulation (Kramer et al., 1996). Elevated levels often indicate increased thrombotic risk and correlate positively with coronary artery disease severity (Chang et al., 2026). Our findings further support its potential as a biomarker for thrombotic events associated with UA. LPC (16:0/0:0), a lysophosphatidylcholine, plays a crucial role in inflammatory responses and apoptosis. Previous studies have demonstrated that this lipid molecule is closely linked to endothelial dysfunction and the progression of atherosclerosis (Liu et al., 2012; Kondreddy et al., 2025). Its significant upregulation in UA patients in this study suggests LPC (16:0/0:0) may participate in disease-related lipid inflammatory regulatory networks, demonstrating research value as a metabolic intervention target.

DL-carnitine, a crucial transporter for fatty acid β-oxidation, plays a significant role in myocardial energy metabolism and mitochondrial function. Numerous studies have demonstrated that DL-carnitine supplementation enhances cardiac function and alleviates ischemic injury (Zhang et al., 2018; Li et al., 2022). This study identified elevated levels of DL-carnitine in patients with UA, which may indicate a reprogramming of energy metabolism in response to myocardial ischemic conditions. The observed elevation in DL-carnitine likely reflects a systemic compensatory response to increased myocardial demand for fatty acid oxidation under ischemic stress. However, this elevated plasma level may paradoxically indicate an overwhelmed or dysfunctional carnitine shuttle system, where impaired enzymatic activity creates a metabolic bottleneck despite high substrate availability. Xia et al. identified ten sphingolipids as potential CAD biomarkers using lipidomics in a study with 24 CAD patients and 12 healthy controls, while Shang et al. found three lipid metabolites with diagnostic potential in 52 myocardial infarction patients and 52 controls. Unlike these studies, our research combines high-precision quantitative lipidomics with machine learning and an independent validation cohort, enhancing marker selection’s objectivity and clinical relevance. We uniquely identify the diagnostic potential of TxB3, LPC(16:0/0:0), and DL-Carnitine for CAD, offering new markers and insights for its metabolic classification and diagnosis.

Nevertheless, several limitations warrant consideration. Firstly, while the sample size suffices for preliminary conclusions, it may be insufficient to fully support generalising these findings to broader populations, particularly given the diversity of unstable angina presentation. Additionally, the exclusive reliance on a single center for patient recruitment may introduce selection bias, and there is no assessment of the intra-individual or inter-day variability of the identified metabolic markers. Furthermore, the study’s inclusion criteria, which only encompassed healthy controls without stable angina pectoris or other coronary diseases, constrain the external validity of the findings. Finally, the cross-sectional design employed precludes inferring causality between the identified metabolic markers and UA, as temporal relationships cannot be established. Future studies requiring larger, multicentre cohorts and longitudinal designs are needed to validate these findings and elucidate potential mechanisms linking metabolic dysregulation to UA. Xia et al. identified ten sphingolipids as potential CAD biomarkers using lipidomics in a study with 24 CAD patients and 12 healthy controls, while Shang et al. found three lipid metabolites with diagnostic potential in 52 myocardial infarction patients and 52 controls (Gao et al., 2022; Shang et al., 2022). Unlike these studies, this study combines high-precision quantitative lipidomics with machine learning and an independent validation cohort, enhancing marker selection’s objectivity and clinical relevance. We uniquely identify the diagnostic potential of TxB3, LPC(16:0/0:0), and DL-Carnitine for UA, offering new markers and insights for its metabolic classification and diagnosis.

This study identified specific metabolic signatures for UA by combining lipidomics with machine learning. Using UPLC-MS/MS technology and thorough statistical analysis, 193 lipid metabolites were found to be significantly different. Notably, TxB3, LPC (16:0/0:0), and DL-carnitine showed diagnostic potential as novel UA biomarkers. In summary, this work offers new insights into lipid metabolism in cardiovascular disease and highlights the potential of metabolic profiling for UA diagnosis, pending confirmation through future multi-center external validation studies.

## Data Availability

The data that support the findings of this study are openly available in China National Center for Bioinformation (https://www.cncb.ac.cn/), reference number OMIX016929.
